# Massive screening of copy number population-scale variation in *Bos taurus* genome

**DOI:** 10.1186/1471-2164-14-124

**Published:** 2013-02-26

**Authors:** Francesco Cicconardi, Giovanni Chillemi, Anna Tramontano, Cinzia Marchitelli, Alessio Valentini, Paolo Ajmone-Marsan, Alessandro Nardone

**Affiliations:** 1 Department for innovation in biological, agro-food and forest systems, University of Tuscia, via de Lellis, Viterbo, 01100, Italy; 2 CASPUR, Inter-University Consortium for Supercomputing in Research, via dei Tizii 6b, Roma, 00185, Italy; 3 Department of Physics, Sapienza University of Rome, P.le A. Moro 5, Rome, 00185, Italy; 4 Zootechnical institute, University Cattolica Sacro Cuore, Piacenza, 29100, Italy; 5 IIT@Sapienza, Center for life nano science, Rome, 00185, Italy; 6 Consiglio per la ricerca e la sperimentazione in agricoltura - CRA-PCM, Animal Production Research Centre, via Salaria 31, Monterotondo, 00015, Italy

**Keywords:** Copy number variants, Structural variations, Cattle, *Bos taurus*

## Abstract

**Background:**

Copy number variations (CNVs) represent a significant source of genomic structural variation. Their length ranges from approximately one hundred to millions of base pair. Genome-wide screenings have clarified that CNVs are a ubiquitous phenomenon affecting essentially the whole genome. Although *Bos taurus* is one of the most important domestic animal species worldwide and one of the most studied ruminant models for metabolism, reproduction, and disease, relatively few studies have investigated CNVs in cattle and little is known about how CNVs contribute to normal phenotypic variation and to disease susceptibility in this species, compared to humans and other model organisms.

**Results:**

Here we characterize and compare CNV profiles in 2654 animals from five dairy and beef *Bos taurus* breeds, using the Illumina BovineSNP50 genotyping array (54001 SNP probes). In this study we applied the two most commonly used algorithms for CNV discovery (QuantiSNP and PennCNV) and identified 4830 unique candidate CNVs belonging to 326 regions. These regions overlap with 5789 known genes, 76.7% of which are significantly co-localized with segmental duplications (SD).

**Conclusions:**

This large scale screening significantly contributes to the enrichment of the *Bos taurus* CNV map, demonstrates the ubiquity, great diversity and complexity of this type of genomic variation and sets the basis for testing the influence of CNVs on *Bos taurus* complex functional and production traits.

## Background

Copy number variants (CNVs) represent a significant source of genomic structural variation. Their length ranges from 100 bp to several Megabases (up to 5 Mb) and they comprise insertions, deletions, and duplications [1-5]. CNVs were initially thought to be only associated to diseases, but genome-wide screenings have clarified that they are ubiquitous and widespread in many animal genomes [6-11].

Recent studies have shown that genomic structural variations (including CNVs) are common among normal and healthy individuals [12-14]. They account for more differences between individuals, in terms of total bases involved, and have a higher per-locus mutation rate than SNPs [15]. Understanding their distribution in the population at large is crucial in order to clarify their role in determining the phenotype and/or disease state [16]. In humans, several studies have attempted to characterize CNVs in populations using data from the International Human HapMap Consortium [1,9,13,17,18], and other reference groups [2,3,16]. These studies have confirmed that CNVs are widespread throughout the genome and show a broad variation in their frequency of occurrence in populations. In addition they are present throughout the genomes of all taxa investigated so far: mammals [19-26], birds [27] and invertebrates [28,29].

CNVs exist in at least two distinct, although non-exclusive, states. *Common CNV* polymorphisms (i.e. frequency > 1%) often with multiple allelic states defined by variations in copy number and/or genomic structure; and *rare CNVs*, that typically lead to deletion or duplication of larger genomic segments and exist in fewer allelic states (i.e., hemizygous or trisomic). These latter classes of CNVs are highly penetrant and short-lived in the population, either occurring *de novo* or persisting for only a few generations and subject to purifying selection [30]. While these structural variations are often benign, they can sometimes influence or even disrupt biological functions. For example CNVs have been identified as causative of a number of human diseases [5,11].

*Bos taurus* is one of the most important domestic animal species worldwide. It is one of the most studied ruminant models for metabolism, reproduction, and disease [31]. Consequently, the understanding of the genetic basis of the differences in productive and functional traits in this species has great economic importance and biological significance. In this context, knowledge of the abundance and distribution of CNVs and of their association with phenotypes are of major interest. However, until now, relatively few studies have investigated CNVs in cattle [32-40], none using a population-wide analysis. Therefore, little is known about how CNVs contribute to normal phenotypic variation and disease susceptibility in cattle, compared to humans and other model organisms.

The recent focus of the research community on the study of single nucleotide polymorphisms (SNPs) to assess genetic variation in cattle have promoted the use of genotyping arrays mapping to thousands of loci throughout the genome (e.g. Illumina BovineSNP50 BeadChip with 54,001 informative SNP probes). This type of array is now easily available to scan thousands of individuals at an affordable cost, allowing CNVs to be investigated on a wide scale. Compared to the higher-density of a comparative genomic hybridization array (CGH arrays), a method that detects copy number changes at the level of 5–10 kb, SNP arrays have the advantage of providing both normalized intensities (Log R ratio – LRR), allelic intensity ratios (B allele frequency – BAF) and a better estimate of the loss of heterozygosity (LOH) making CNV detection more robust. Several algorithms are able to detect CNVs using the intensity of fluorescent signals from SNP arrays. In this study we applied the two most commonly used and efficient ones [41], as implemented in the QuantiSNP [42] and PennCNV [43] software, to investigate the genome-wide characteristics of CNVs in five *Bos taurus* breeds. We scanned the 29 autosomal chromosomes in a panel of 2654 animals and identified 4830 unique CNV candidates belonging to 326 regions, comparing our findings with existing publicly available information on cattle CNVs and investigated the identity and function of genes located within the duplicated regions. Our results significantly enrich the current knowledge about copy-number variants in the *Bos taurus* genome determining their distribution across the genome in five dairy and beef cattle breeds (Italian Friesian, Italian Brown, Italian Simmental, Marchigiana and Piedmontese). These findings are an important resource for follow-up studies on cattle genome structure and CNV-trait association [44,45].

## Results

### CNV discovery and distribution

**Figure 1:**
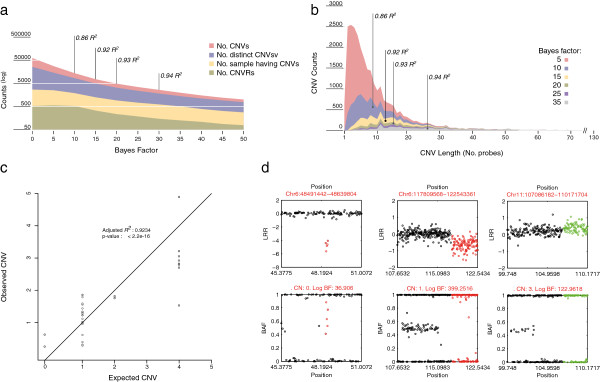
**Validation steps and CNV profiles as a function of different Bayes factor thresholds.** (**a**) Density plot of the number of redundant and non-redundant CNVs in samples bearing CNVs and (**b**) of the CNV regions for each CNV as a function of the indicated thresholds. Vertical lines indicate the adjusted R^2^ values with the different Bayes factor (BF) thresholds (BF/R^2^;10/0.86; 15:0.92; 20/0.93; 30/0.94). (**c**) Correlation between the number of CNVs and the results of qRT-PCR experiments (p0.0001; R^2^: 0.9234). (**d**) Log R ratio (LRR) and B allele frequency (BAF) plots of three copy-number variants (CN: 0, position: Chr6:48491442–48639804; CN: 1, position:Chr6:117809568–122543361; CN: 3, position: Chr11:107086182–110171704).

After dataset cleaning, a total of 51582 SNPs from the BovineSNP50 BeadChip were independently analysed with QuantiSNP [42] and PennCNV [43] to identify cattle CNVs. After CNV calling, we identified the best Bayes Factor (BF) threshold to be used by plotting the number and length of discovered CNV as a function of the Bayes factor values, and used the adjusted R2, obtained by qRT-PCR (see Methods and Materials section) as a measure of the false positive rate. Since in the literature [44,46,47] a BF threshold values of 10 is very often used and there is no evident improvement in the R2 value for BF values higher than 15, we assumed 15 as the best value that minimizes false positive calling rate and maximizes CNV calling number [48,49], thus obtaining a good confidence also for single-observed CNVs (Figure 1). As expected and as shown in Figure 1a and 1b, the proportion of CNV length classes detected changes as a function of BF. BF measures the confidence we have in the CNV and depends upon signals arising from a number of contiguous probes. Short CNVs detected by fewer probes result with low BF values, and consequently longer CNVs detected by more probes result in higher BF values. The somewhat larger than usual BF value used here therefore is unfavorable to short CNVs. By setting a high BF value we preferred to identify a lower number of short CNVs but highly confident. It should be noted, however, that the skew of distribution observed in Figure 1b is consistent with several studies reported in the literature [1,30,35].

**Figure 2:**
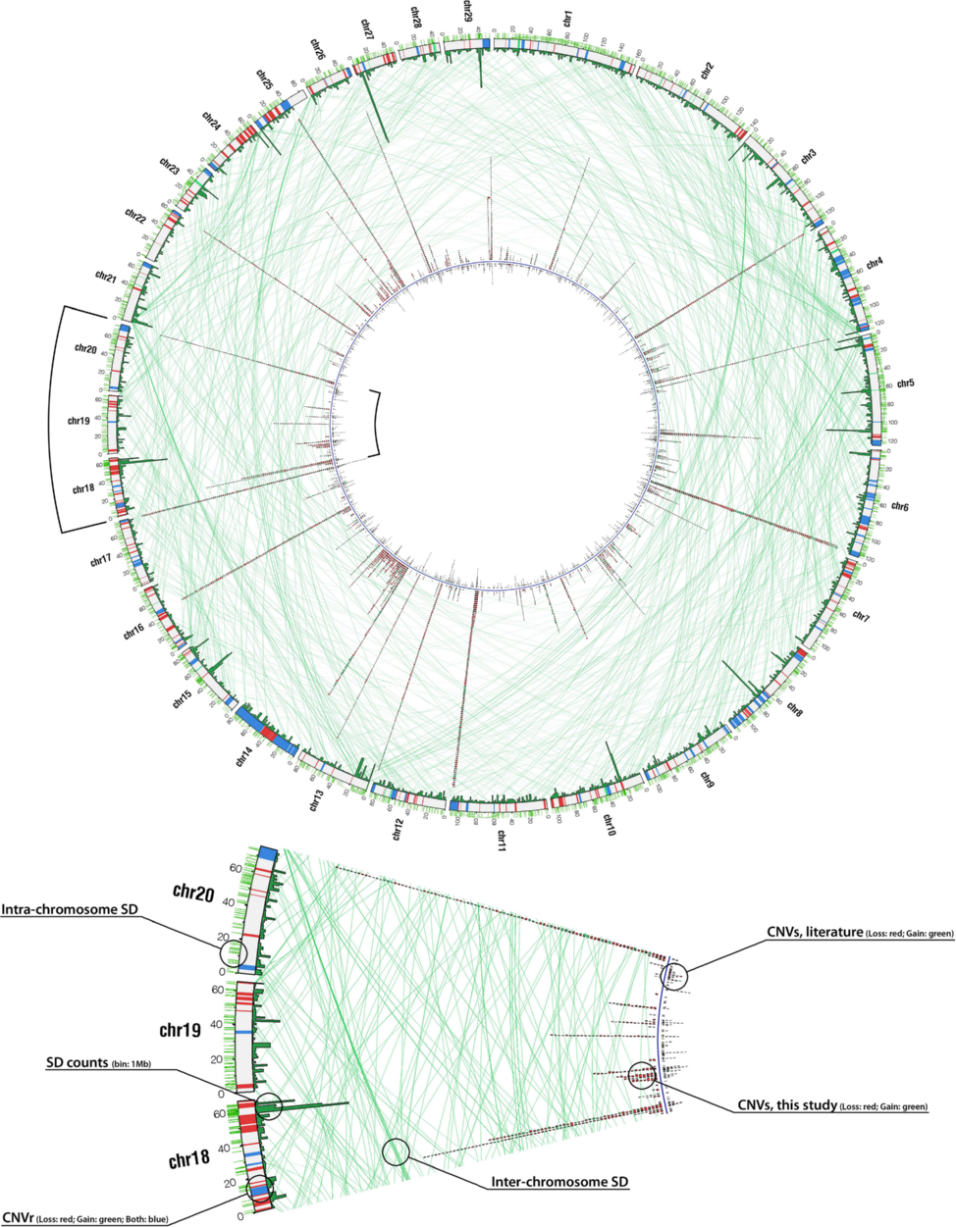
**Comprehensive circular map of autosomal copy-number variants and segmental duplications in*Bos taurus.*** From the outside to the inside of the external circle: Chromosome name; genomic location (in Megabases); lines linking SD positions within each chromosome; bars depicting the CNV regions (loss in red, gain in green and both in blue); histogram representing the number of observed SD in the corresponding position (each bin is 1 Megabase). Light green lines link positions of SD between different chromosomes. The internal blue circle is flanked by red and green tiles representing the loss and gain events identified in this study (directed towards the outside) and in previous studies (towards the inside).

A total of 2654 individuals from five breeds were analysed. We identified 7493 CNVs (4839 after eliminating redundancy) (Figure 2; Additional file 1: Table S1) and 402 CNV regions (CNVRs) (Additional file 2: Table S2) determined by aggregating overlapping CNVs across all samples.

Each individual possesses an average of 6 CNVs, ranging from 23kb to 4963kb with mean and median length of 930 kb and 700 kb, respectively. CNV regions (CNVRs) include 18 CNVs on average and span regions with length between 53kb to 10552kb, with mean and median length of 1240kb and 782kb, respectively. Furthermore, 37 CNVRs have an observed frequency >1%, 24 a frequency > 2% and 5 a frequency > 5%. Considering all 7493 CNVs, 92 of them (1.22%) are homozygous deletions, 5259 (70.18%) heterozygous deletions, 1592 (21.25%) and 550 (7.35) are duplications with three and four copies respectively (Table 1). We observed on average 258 CNVs per chromosome, a significant fraction of which (10%) located in BTA6 (*Bos taurus* autosome 6) chromosome, while the lowest number of CNVs (0.3%) was in BTA28.

**Table 1:** CNV statistics

	**Total number**	**Average number per sample**	**Average size of CNVs (kb)**	**Median size of CNVs (kb)**	**No. of common CNVs (freq> 1%)**	**2%**	**5%**
Distinct CNV	4830	6.09	931	696	290	80	14
CNV regions	402	4.52	1240	782	37	24	10
	No. of Gain	No. of Loss	No. homo/heterozygous Loss		No. of Both	Ration (Loss/Gain)	Genes
Distinct CNV	2142	5351	92	5259	2663	2.50	-
CNV regions	140	366	-	-	126	2.61	5789

Summary of the identified copy-number variants in the five analysed *Bos taurus* breeds.

Eleven copy-number variation regions of homozygous and heterozygous deletions and duplications (Additional file 2: Table S2) were validated by quantitative real-time PCR. These were randomly selected across eleven autosomal chromosomes. Each CNV was amplified in a minimum of three and a maximum of seven specimen belonging to different breeds, for a total of 50 validation tests. The CNV copy number estimated by qRT-PCR was plotted against the BeadChip copy number determination (Figure 1c). Linear regression analysis showed a high level of correlation (R^2^ = 0.92) and a curve slope of 1.00 (Standard Error: 0.05; *p*-value = 2.2e-16).

The analysis of the distribution of CNV size indicates that with the BF values used less than 2% of CNVs are ≤ 100kb, 12% have a length between 100 and 250kb, 27% have a length between 250 and 500kb, 33% have a length between 500 and 1000 kb, and 25% are longer than 1 Mb. In few samples we identified CNVs about 8Mb long. CNVR number and length are not significantly correlated to chromosome length. BTA29 hosts three CNVRs, while BTA6, has 20 CNVRs, the highest value. Out of the 326 CNVRs, 192 include loss-only events, 31 gain-only events and 103 include both. Loss events are approximately 6.2-fold more common than gain events in CNVRs, while the corresponding rate is 2.5-fold for CNVs. CNVRs affected by loss events have, on average, smaller size than gain regions, in line with the recent published results of Hou et al. [37].

**Figure 3:**
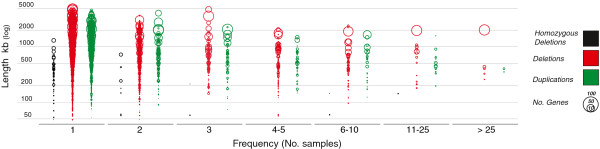
**Copy-number variant length, gene content and frequency distributions.** CNVs (circles) plotted according to their copy-numbers (colours), length (y-axis), frequency in the population (x-axis, expressed as number of individuals having that CNV), and numbers of affected coding genes inside each CNV (circle size).

Looking at the genomic distribution of CNVs within the population, they collectively span a wide fraction of the genome, ~20% of the autosomal genome (497 Mb), in line to what has been found in humans (~16%) [30]. These findings prove that potentially significant portions of the genome can vary in number. There is a substantial difference in the fraction of the genome affected by common (defined as more frequent than >1%) and rare CNVs. The common ones occupy only ~0.1% of the genome suggesting that the bulk of the observed copy-number variations belong to the rare CNV set. There is also a different frequency distribution among CNV types (gain or loss). Duplications and heterozygous deletions are substantially retained in the population while homozygous deletions are found only at very low frequency, generally in one or two samples. These findings suggest the existence of purifying selection in the population due to the potentially deleterious effect of homozygous deletions (Figure 3; Additional file 3: Table S3).

### CNV association with segmental duplications and gene content

Although the complete set of mechanisms responsible for generating CNVs is unknown, studies on cattle [2,37] and other mammalian species [5,29,40] highlighted an enrichment of CNVs near segmental duplications (SD). Segmental duplications, defined as genomic regions of high sequence identity (greater or equal to 90%) to more than one genomic locus, may mediate CNV genesis by acting as a substrate for non-allelic homologous recombination. These recombination events may result in amplification, deletion, inversion, or copy number variants. We tested whether there is a non-random association between the CNVs that we discovered and known SD regions [45] and found a significant overlap: 76.7% of the CNVs intersect with SDs (*p*-value < 0.001 as estimated by a random permutation test).

The 4839 non-redundant CNVs found within autosomes overlap with a total of 5789 known genes (Additional file 4: Table S4 and Additional file 5: Table S5). Among them, 5019 (87%) are protein coding genes, 676 (12%) non-coding RNAs (229 miRNA, 73 rRNA, 211 snRNA, 131 snoRNA, 32 misc_RNA), and 94 (1%) are pseudogenes and retrotransposable elements. The ~5000 loci included in CNVs contain about 25% of the estimated total number of genes of the species (Additional file 4: Table S4). This fraction is higher than what has been reported in similar papers (Hou *et al.*, 1,263 [37], Bae *et al.*, 538 [34]) but comparable with the results of the population-scale study in humans carried out by Mills and colleagues [18], who mapped genomic structural variations affecting more than 10000 genes.

We used the DAVID tool [50] to analyse the Gene Ontology (GO) functional categories of the protein coding genes located in CNVs (Table 2). Several GO terms were found to be significantly over-represented (*p*-adjusted < 0.05). The most enriched GO cellular component categories among the protein coding genes are related to ribosomal activity, with an enrichment fold larger than two (cytosolic small ribosomal subunit, 3.43; cytosolic ribosome, 3.2; small ribosomal subunit, 2.43; ribosomal subunit, 2.06). This set of genes has a limited spectrum of functions, with one-third of their GO terms being related to metabolism. This is also confirmed by a KEGG pathway enrichment analysis (Table 2). We found a significant enrichment (~2-fold) in Nitrogen metabolism, Ribosomal and Oxidative phosphorylation pathways. Interestingly, the same conclusion has been reached in a recent study of CNVs with next-generation sequencing in cattle [40], thus suggesting that CNVs may contribute to the genetic variance of production traits in this species.

**Table 2:** KEGG pathway and Gene ontology enrichment

**Category**	**Term**	**Count**	**Fold Enrichment**	**PValue**	**Benjamini**	
GOTERM_CC_FAT	GO:0022627~cytosolic small ribosomal subunit	11	3.43	0.0002	0.0140	
GOTERM_CC_FAT	GO:0022626~cytosolic ribosome	11	3.20	0.0004	0.0222	
GOTERM_CC_FAT	GO:0015935~small ribosomal subunit	15	2.43	0.0010	0.0401	
GOTERM_CC_FAT	GO:0033279~ribosomal subunit	24	2.06	0.0004	0.0247	
GOTERM_CC_FAT	GO:0044448~cell cortex part	22	2.00	0.0010	0.0408	
GOTERM_CC_FAT	GO:0005874~microtubule	38	1.66	0.0009	0.0423	
GOTERM_CC_FAT	GO:0005840~ribosome	67	1.45	0.0008	0.0389	
GOTERM_CC_FAT	GO:0005829~cytosol	87	1.36	0.0011	0.0403	
GOTERM_CC_FAT	GO:0044429~mitochondrial part	118	1.35	0.0002	0.0158	
GOTERM_CC_FAT	GO:0030529~ribonucleoprotein complex	106	1.33	0.0008	0.0417	
GOTERM_CC_FAT	GO:0005739~mitochondrion	219	1.31	0.0000	0.0014	
GOTERM_CC_FAT	GO:0043233~organelle lumen	173	1.30	0.0000	0.0065	
GOTERM_CC_FAT	GO:0031974~membrane-enclosed lumen	180	1.30	0.0000	0.0094	
GOTERM_CC_FAT	GO:0070013~intracellular organelle lumen	172	1.30	0.0001	0.0070	
GOTERM_CC_FAT	GO:0031981~nuclear lumen	125	1.28	0.0012	0.0421	
GOTERM_CC_FAT	GO:0043228~non-membrane-bounded organelle	316	1.20	0.0000	0.0081	
GOTERM_CC_FAT	GO:0043232~intracellular non-membrane-bounded organelle	316	1.20	0.0000	0.0081	
KEGG_PATHWAY	bta03010:Ribosome	41	2.00	0.0000	0.0005	
KEGG_PATHWAY	bta00190:Oxidative phosphorylation	52	1.64	0.0001	0.0097	
Category enrichment for Italian Simmental						
**Category**	**Term**	**Count**	**Fold Enrichment**	**PValue**	**Benjamini**	**Genes**
GOTERM_CC_FAT	GO:0005576~extracellular region	10	6.51	0.0000	0.0000	LOC751562; PRP1,3,6,9; LOC751563; CSH2; PRP-VII; PRL
GOTERM_MF_FAT	GO:0005179~hormone activity	10	82.73	0.0000	0.0000	LOC751562; PRP1,3,6,9; LOC751563; CSH2; PRP-VII; PRL
INTERPRO	IPR001400:Somatotropin hormone	10	509.50	0.0000	0.0000	LOC751562; PRP1,3,6,9; LOC751563; CSH2; PRP-VII; PRL
INTERPRO	IPR018116:Somatotropin hormone, conserved site	9	429.89	0.0000	0.0000	LOC751562; PRP1,3,9; LOC751563; CSH2; PRP-VII; PRL
INTERPRO	IPR012351:Four-helical cytokine, core	7	172.57	0.0000	0.0000	LOC751562; PRP1,3,4,6; CSH2; PRL
PIR_SUPERFAMILY	PIRSF001825:prolactin/lactogen/growth hormone	7	302.56	0.0000	0.0000	LOC751562; PRP1,3,4,6; CSH2; PRL
SP_PIR_KEYWORDS	hormone	10	106.05	0.0000	0.0000	LOC751562; PRP1,3,6,9; LOC751563; CSH2; PRP-VII; PRL
SP_PIR_KEYWORDS	Secreted	5	5.73	0.0056	0.0258	CSH2; PRP1,3,4; PRL
SP_PIR_KEYWORDS	signal	6	4.18	0.0054	0.0370	PRP1,3,4,6; CSH2; PRL
UP_SEQ_FEATURE	disulfide bond	5	5.27	0.0040	0.0258	CSH2, PRP1, PRP4, PRL, PRP3
UP_SEQ_FEATURE	signal peptide	6	5.03	0.0007	0.0095	PRP1,3,4,6; CSH2; PRL

KEGG pathway and Gene ontology (GO) categories significantly over represented, with false discovery rate (Benjamini) and gene lists (Ensemble gene ID).

**Figure 4:**
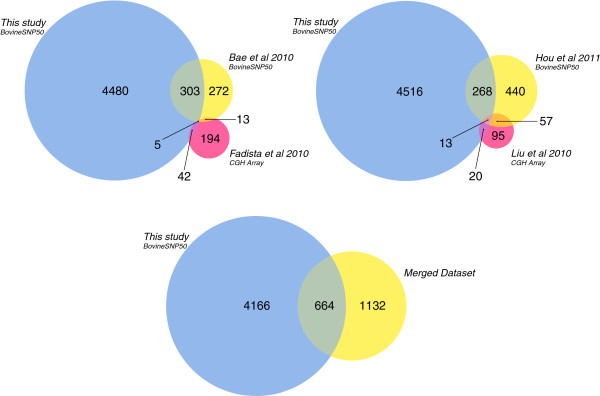
**Comparisons between the CNVs discovered in this study and other known CNVs.** Venn diagrams showing the comparison between the non-redundant CNVs (4839) detected in this study and the available CNV datasets (Bae et al. 2010; Hou et al. 2011; Fadista et al. 2010; Liu et al. 2010) and with their union (Merged Dataset).

Figure 4 shows the comparison of our data with those obtained in similar studies available in the literature [34,35,37,51]. The four studies we considered used different approaches and different breeds and altogether detected 1810 CNVs from less than 1000 samples. Among them, the two studies based on the same genotyping array we used (BovineSNP50 v1) (Bae et al. [34] and Hou et al. [37]) respectively detected 308 and 281 CNVs overlapping with those described here. These correspond to 52% and 36% of the CNVs detected in our study.

The other two datasets obtained by Fadista *et al*. [35] and Liu *et al*. [51] who used a CGH array, show a more limited overlap with our dataset, namely 19% and 18%. The lower overlap in these cases is very likely due to the fact that the CGH array they used has a much higher density of probes (420 bases of average probe spacing [35]) compared to the BovineSNP50 beadchip (49 kb of average probe spacing). The identification with high confidence of short CNVs (< 50 kb), even the more frequent ones [35,40], is much harder with the Illumina genotyping chip, which identifies CNVs having a distribution skewed towards large size. We also measured the percentage of overlap of the CNVs detected by us and by two other studies based on the next-generation sequencing approach [39,40]. Even though the authors of these studies examined fewer samples (two samples in [39] and six in [40]), their more accurate methodology, at nucleotide resolution, shows a moderately higher overlap with our data (33% and 22% respectively, Additional file 1: Table S1). The only partial overlap of the CNVs we find with those detected in other studies can, in principle, be explained by the different breeds used here. Many CNVs appear to be breed specific and may contribute to breed differentiation. On the other hand several studies [30] suggest that the bulk of CNV variability is more individual than breed specific and therefore the larger number we find is most likely due to the fact that we tested a large number of individuals.

### *Bos taurus* CNV features among breeds

We looked at the differences among the five *Bos taurus* breeds investigated: Italian Friesian (dairy), Italian Brown (dairy), Italian Simmental (dairy/beef), Piedmontese (beef), and Marchigiana (beef).

**Figure 5:**
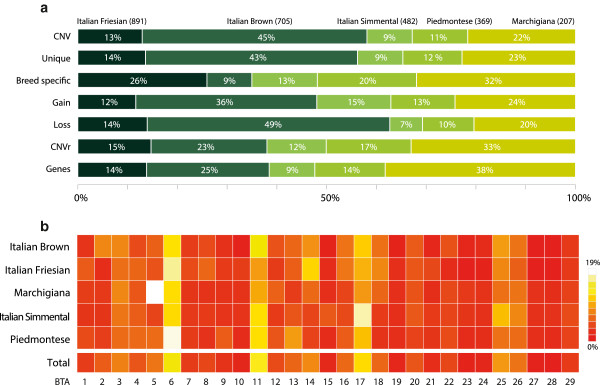
**CNV breed profiles. a**) Stacked bar chart showing the percentage of CNVs (total, unique, specific, gain and loss CNVs), CNVRs and number of genes affected for each breed studied with respect to each sample size. The number of samples for each breed is shown in parenthesis. **b**) Heatmap showing the CNV distribution in the 29 autosomal chromosomes. Dark red tiles represent low frequency CNVs, while pale yellow/white indicate high frequency CNVs.

Among them, the Italian Brown shows the higher abundance of unique, single CNVs and CNVRs (Table 3, Figure 5a) (*p*-value < 0.0001), while Marchigiana and Italian Friesian have a higher number of single and unique CNVs than the Piedmontese and Italian Simmental (*p*-value < 0.001). The Italian Brown shows the highest rate of loss events (*p*-value < 0.0001), while the Piedmontese shows the lowest frequency of deletion events per sample (*p*-value < 0.01). The Italian Brown and Marchigiana have, on average, significantly more gain events (*p*-value < 0.0001) than Italian Friesian and Italian Simmental, but not more than Marchigiana and Piedmontese, probably due to the wider distribution of the latter. While Italian Simmental has significantly less gain events than all breeds but Italian Friesian (*p*-value < 0.0001). When considering the average proportion of single CNVs per CNVRs (CNV density) within each breed, it can be observed that the Italian Brown has a more concentrated distribution (more CNVs per CNVRs), two times less sparse than the Italian Simmental, the Piedmontese and the Marchigiana (p-value < 0.006). We found no significant difference in the distributions of CNV lengths among breeds, with the only exception of the Italian Simmental that shows a moderately lower mean and median lengths. The average number of CNVs per sample is comparable among the five breeds.

**Table 3:** **CNV events by* Bos taurus *breeds**

**Breed**	**No. samples**	**CNV counts**		**CNV unique**		**CNV specific**		**Gain**		**Loss**		**CNVR count**		**Protein coding genes**		**Total length (Mb)**	**Mean length (kb)**	**Median (kb)**	**Min length (kb)**	**Max length (kb)**
Italian Friesian	891	1522	*1.71*	1179	*1.32*	1151	*1.29*	419	*0.47*	1146	*1.29*	169	*0.19*	3056	*3.43*	323.84	8.04	5.79	0.23	49.57
Italian Brown	705	4198	*5.95*	2923	*4.15*	316	*0.45*	1034	*1.47*	3164	*4.49*	211	*0.30*	4310	*6.11*	441.94	8.45	6.25	0.48	49.63
Italian Simmental	482	578	*1.20*	427	*0.89*	316	*0.66*	289	*0.60*	292	*0.61*	74	*0.15*	1094	*2.27*	108.36	6.30	4.65	0.49	43.74
Piedmontese	369	543	*1.47*	427	*1.16*	366	*0.99*	192	*0.52*	351	*0.95*	81	*0.22*	1295	*3.51*	95.75	7.29	6.01	0.61	46.30
Marchigiana	207	591	*2.86*	459	*2.22*	329	*1.59*	202	*0.98*	389	*1.88*	88	*0.43*	1964	*9.49*	140.23	7.86	7.86	0.25	44.74
No Samples	Marchigiana %	Piedmontese %	Italian Simmental %	Italian Friesian %	Italian Brown %															
1	91.07	88.76	84.31	87.56	81.66															
2	5.88	7.73	10.07	7.83	10.71															
3	1.53	1.17	2.34	2.39	3.59															
4-5	0.65	0.94	1.41	0.82	2.26															
6-10	0.65	0.70	1.17	0.82	1.09															
10-25	0.00	0.70	0.70	0.58	0.58															
>25	0.22	0.00	0.00	0.00	0.10															
No Samples	Marchigiana	Piedmontese	Italian Simmental	Italian Friesian	Italian Brown															
1	418	379	360	1063	2387															
2	27	33	43	95	313															
3	7	5	10	29	105															
4-5	3	4	6	10	66															
6-10	3	3	5	10	32															
10-25	0	3	3	7	17															
>25	1	0	0	0	3															

Summary of the identified copy-number variants in each of the five *Bos taurus* breeds. Numbers in italics are normalized by sample counts.

The CNVs distribution among chromosomes (Figure 5b) is, in general, homogeneous and consistent across breeds with the exception of two breeds showing a peak in CNV frequencies in two different chromosomes (BTA5, BTA17). In BTA5 the percentage of CNVs in four breeds is only 3.4% (*p*-value < 1e-12), while in Marchigiana this chromosome carries 18.1% of all its CNVs observed (107/591 CNVs). The same is true for the BTA17 where the Italian Simmental has 18.5% of the CNVs (107/578 CNVs) to be compared with 7.8% for the other breeds (*p*-value < 0.04). Considering all the other CNV features (length, population frequency and chromosome position), no significant difference was observed among breeds. Overall this findings also suggest that differences between individuals seems to be much larger than differences between breeds.

Gene ontology enrichment was computed taking into account the genes involved in CNVs for each breed. Only the 17 genes of the Italian Simmental (Additional file 6: Table S6, Additional file 7: Table S7) showed functional enrichment (Table 2). In particular we observed a significant enrichment for GO term involved in Somatotropin and prolactin/lactogen/growth activity genes caused by a single and breed-specific CNV (chr23:33,906,415-36,330,036; three copies) that contains 12 loci (LOC751562-3, PRP1,3,4,6,9, CSH2, PRP-VII, PRL, HDGFL1, MIR2284C). These genes belongs to the PRL family (prolactin related proteins), expressed in the placenta around the first 60 days of gestation and are involved in the establishment and maintenance of pregnancy [52]. Prolactin genes (PRL) are known to have undergone rapid evolution in the lineage leading to ruminants [51-54] and to be duplicated in all well studied ruminants species. The evidence presented here suggests a possible implication of this cluster in the explanation of genetic variation of production traits.

## Discussion

In this investigation we find more CNVs than in previous studies [34-36,39,40,51]. This is likely due to the large number of individuals analysed. There is also a (probably less relevant) difference in the analysis tools that we have used, PennCNV (as in previous studies) and QuantiSNP, known to be more efficient [41]. Given the high number of individuals analysed we detected a number of previously unidentified rare CNVs. It has been reported that in humans, for example, the bulk of the observed copy-number variation is present at ~0.02%–1% frequency [30].

We cannot exclude the presence of false positives in our dataset, but the results of qRT-PCR validation of 50 individuals for the presence of 11 CNVs (see Figure 1c, R2 = 0.92) suggests that the level of BF (BF = 15 vs the commonly used threshold of 10) used in favour of the detection of false positive CNVs was rather effective. Only the validation reported by Fadista et al. [35] is comparatively equally extensive (65 individuals and 6 CNVs). Furthermore, the number of CNVs per individual in our case averages of 2.8, a lower value than what found in other studies (around 3.6 in *Bos taurus* with the same SNP chip). We are therefore confident that the rate of false positives we detected is reasonably low and that do not affect the overall picture.

Notwithstanding the high number of samples examined and CNVs identified, we likely still haven't drawn a complete picture of CNV presence in cattle, mainly because of the limitations of the genotyping array used. We are well aware that the relatively low density of the Illumina arrays with respect of other methods (CGH arrays, whole re-sequencing) make the detection of short CNVs very hard, while it is very well documented, by deep-sequencing methodologies that in *Homo sapiens*[18,55] and more recently in *Bos taurus* the most populated class of CNVs is that of variants shorter than 50 kb [39,40]. This limitation will only be partially overcome by using the more recent higher-density BovineHD BeadChip (777 k SNPs). This chip, with its 3430 bp average probe distance is ~8 times less dense than the available CGH arrays and therefore would not solve the problem of incompleteness. It is unlikely that any single available technology will capture all genome structural variations and the use of multiple experimental methods (sequence assembly comparisons, paired-end sequencing, sequencing analysis and high-resolution tiling arrays) will be needed to unravel the complexity of genome variations.

## Conclusion

Our study presents the first population-scale description of copy number variants in *Bos Taurus* obtained by analysing data from more than 2500 individuals belonging to five different dairy and beef breeds and using two different bioinformatics algorithms. We found that CNVs collectively span ~20% of the genome and that a significant portion of the genome is potentially subject to variation in copy number, as observed in humans. We described here the frequencies, patterns, and the potential of gene landscape impact of such cattle-specific and breed-specific CNVs. Many CNVs include genes having specific biological roles, e.g. in metabolism, and are thus likely to be functional. Our population scale analysis reveals that, because of their very low frequency, many CNVs are likely to arise independently, generating increased diversity among individuals and providing insight into the penetrant behaviour of CNVs in the population. This cattle CNV map provides information that complements SNP information and may be added to SNP-based genome-wide association and selection studies. A more comprehensive knowledge of the full landscape of bovine genetic variation permits a better understanding of ruminant biology and a further improvement of selection methods in this species.

## Methods

### Ethics statement

Animal handling and DNA extraction was carried out following national guidelines and was approved by the animal ethics committee.

### Systematic genome-wide CNV analysis

We studied CNVs in a sample of 2654 Italian bulls (*B. taurus* males used for reproductive purposes in Italian breading). The selection of only bulls is due to the fact that males are usually the ones screened for genotyping and genetically evaluated to record the production traits of their offsprings. The animals belong to five different breeds (891 Italian Friesian, 705 Italian Brown, 482 Italian Simmental, 369 Piedmontese, 207 Marchigiana). Genomic DNA of all samples was analysed using the BovineSNP50 v1 BeadChip 54001 probes (Illumina, San Diego, CA) [56] according to the standard protocol [57]. Sex chromosomes were excluded from the analysis and only autosomes were used. The QuantiSNP [42] and PennCNV [43] tools were used to identify copy number deletions and duplications. Both methods are based on a Hidden Markov Model for the detection of CNVs from Illumina high-density SNP genotyping data. PennCNV is the most frequently used algorithm for CNV studies of this type, partly because of the user-friendly design of the program. Its low false positive rate is another convenient aspect. By contrast, QuantiSNP outperformed six other methods in a recent evaluation study of CNV calling algorithms [41]. We deemed the combined use of both algorithms to be a valid strategy.

Samples with LogR ratio (the normalized total intensity at each locus) higher than 0.30 were filtered out together with individuals with CNV longer than 8Mb, likely to be affected by diseases [58]. For both QuantiSNP and PennCNV, a quality control step for GC-content was performed to check for GC-wave factor and subsequently taken into account for correcting the bias in the analysis [59]. To optimally tune the parameters, such as GC wave factor correction, a training dataset composed of 10% of the data was used. Next, a quality filter for CNV calling based on Bayes Factor thresholds using parameters reported previously [44-47] was applied followed by quantitative PCR (qRT-PCR). The qRT-PCR was used to select the BF threshold with the lower false positive rate. When both the QuantiSNP and PennCNV algorithms detected overlapping CNVs, those with higher BF were selected. All statistical tests to estimate differences in CNV features among breeds, were performed using the Wilcoxon-Mann–Whitney rank sum test statistic as implemented in the R package (wilcox.test, http://www.r-project.org).

### Association between CNV, segmental duplication and gene content

The non-random association between CNVs and segmental duplications was tested by determining the direct overlap of CNV boundaries with the segmental duplication location available from the literature [45]. The association test was performed by comparing the data with those obtained by randomly selecting a segment length from the distribution of CNV lengths and a valid chromosomal location for 1000 times.

Gene content of the cattle CNV regions was obtained via the Ensemble BioMart tool [60] using the genome version Btau_4.0. The obtained list of protein coding genes was used to determine the GO terms and pathway enrichment using the DAVID Bioinformatics resource [50]. The Benjamini method for multiple testing correction was used [61].

### CNV validation

To validate the discovered CNVs, TaqMan quantitative real-time PCR was performed on 50 individuals in 11 regions (Additional file 1: Table S1). Reactions were performed in triplicate in a volume of 25 μl with the Maxima Probe qPCR master mix (Fermentas) on a LightCycler® 480 System (Roche). The PCR cycling conditions were: pre-incubation for 15 min at 95°C, 55 cycles of 15 s at 95°C, 30 s at 58°C. The PCR products were also sequenced to verify the correctness of the amplification region. Primer efficiency was tested for each primer pair (Additional file 1: Table S1) over five dilution points using Maxima SYBR Green qPCR master mix (Fermentas). BTF3 was used as reference gene for all qPCR experiments as in Bae et al. 2010. The quantification analysis was performed using the R package qpcR (http://www.dr-spiess.de/qpcR.html) using the ΔΔCt method [21,62]. The Regression analyses were calculated with the linear model fit function (lm) implemented in R (http://www.r-project.org).

## Abbreviations

BAF: B-allele frequency; BF: Bayes factor; BTA: *Bos taurus* autosome; CGH: Comparative genomic hybridization; CNV: Copy number variation; CNVR: Copy number variation region; GO term: Gene Ontology term; LRR: Log R ratio; miRNA: MicroRNA; qRT-PCR: Quantitative real-time polymerase chain reaction; rRNA: Ribosomial RNA; SD: Segmental duplication; snoRNA: Small nucleolar RNA; SNP: Single nucleotide polymorphism; snRNA: Small nuclear RNA

## Competing interests

The authors declare that they have no competing interests.

## Authors’ contributions

FC, CM and AN conceived and designed the project. FC and GC carried out all the bioinformatics analysis under the supervision of AT and AV. FC and CM carried out the qRT-PCR experiments. FC, GC, AT, PAM wrote the manuscript. All the authors have read and approved the manuscript for publication.

## Supplementary Material

Additional file 1 **CNVs dataset.** Complete list of CNV found in this study. Click here for file

Additional file 2 **CNVRs dataset.** Complete list of CNVR found in this study. It also includes the list of the types of CNV in each region. Click here for file

Additional file 3 **CNV distribution.** List of CNVs and their copy number, length, frequency in the population and number of genes included. Click here for file

Additional file 4 **CNV gene content.** List of genes in each copy number variant. Click here for file

Additional file 5 **Gene list.** Complete list of the involved genes. Click here for file

Additional file 6 **Breed specific genes.** Complete list of genes specific for each of the five studied *Bos taurus* breeds. Click here for file

Additional file 7 **Gene frequency.** Complete list of genes and their frequency for each *Bos taurus* breed. Click here for file
